# Neuroprotective Effect of Quercetin Against the Detrimental Effects of LPS in the Adult Mouse Brain

**DOI:** 10.3389/fphar.2018.01383

**Published:** 2018-12-11

**Authors:** Amjad Khan, Tahir Ali, Shafiq Ur Rehman, Muhammad Sohail Khan, Sayed Ibrar Alam, Muhammad Ikram, Tahir Muhammad, Kamran Saeed, Haroon Badshah, Myeong Ok Kim

**Affiliations:** Division of Applied Life Science (BK 21), College of Natural Science, Gyeongsang National University, Jinju, South Korea

**Keywords:** lipopolysaccharide, natural flavonoids, quercetin, activated gliosis, neuroinflammation, neurotoxicity, memory performance

## Abstract

Chronic neuroinflammation is responsible for multiple neurodegenerative diseases, such as Alzheimer’s disease, Parkinson’s disease, and Huntington’s disease. Lipopolysaccharide (LPS) is an essential component of the gram-negative bacterial cell wall and acts as a potent stimulator of neuroinflammation that mediates neurodegeneration. Quercetin is a natural flavonoid that is abundantly found in fruits and vegetables and has been shown to possess multiple forms of desirable biological activity including anti-inflammatory and antioxidant properties. This study aimed to evaluate the neuroprotective effect of quercetin against the detrimental effects of LPS, such as neuroinflammation-mediated neurodegeneration and synaptic/memory dysfunction, in adult mice. LPS [0.25 mg/kg/day, intraperitoneally (I.P.) injections for 1 week]-induced glial activation causes the secretion of cytokines/chemokines and other inflammatory mediators, which further activate the mitochondrial apoptotic pathway and neuronal degeneration. Compared to LPS alone, quercetin (30 mg/kg/day, I.P.) for 2 weeks (1 week prior to the LPS and 1 week cotreated with LPS) significantly reduced activated gliosis and various inflammatory markers and prevented neuroinflammation in the cortex and hippocampus of adult mice. Furthermore, quercetin rescued the mitochondrial apoptotic pathway and neuronal degeneration by regulating Bax/Bcl2, and decreasing activated cytochrome c, caspase-3 activity and cleaving PARP-1 in the cortical and hippocampal regions of the mouse brain. The quercetin treatment significantly reversed the LPS-induced synaptic loss in the cortex and hippocampus of the adult mouse brain and improved the memory performance of the LPS-treated mice. In summary, our results demonstrate that natural flavonoids such as quercetin can be beneficial against LPS-induced neurotoxicity in adult mice.

## Introduction

Inflammation is a biological response initiated by various types of tissue upon sensing any foreign particle; the purposes of the response are to prevent further tissue harm and injury, to clear and repair damaged tissue, and to eliminate pathogenic elements. However, if inflammation is prolonged, then it becomes chronic inflammation and leads to progressive degeneration. The central nervous system (CNS) contains glial cells, including astrocytes and microglia, that serve as an immune system for the CNS, defending it against pathogens and maintaining the normal structure of neurons (Witte et al., 2010; Badshah et al., 2015b). Tissue damage and systemic inflammation lead to glial cell activation, which releases inflammatory mediators and induces inflammatory diseases in the brain, such as meningitis and multiple sclerosis, as well as non-inflammatory diseases, such as Alzheimer’s disease (AD), Parkinson’s disease (PD) and Huntington’s disease (HD) (Qin et al., 2007; Di Filipopo et al., 2010; Tajuddin et al., 2014). Numerous studies have reported that the activation of glial cells releases harmful mediators such as reactive oxygen species (ROS), nitric oxide, cytokines and inflammatory mediators, which ultimately lead to neuroinflammation-mediated neuronal degeneration (Di Filippo et al., 2008; Chen W.W. et al., 2016; Kempuraj et al., 2016, 2017). Lipopolysaccharide (LPS) is an essential component of the cell wall of gram-negative bacteria and acts as a potent stimulator of immune cells, including glial cells, inducing the expression of proinflammatory cytokines (Sheng et al., 2003; Biesmans et al., 2013). Various *in vitro* and *in vivo* studies have reported that LPS activates glial cells, leading to neuroinflammation followed by neurodegeneration (Johansson et al., 2014; Qin et al., 2015; Khan et al., 2017).

Flavonoids are a large group of natural polyphenolic plant pigments that are ubiquitous in many commonly consumed vegetables, fruits, grains, herbs, and beverages. Flavonoids have shown many forms of bioactivity, such as anticancer, cardiovascular, antioxidant, neuroprotective, and anti-inflammatory properties (Mennen et al., 2004; Yao et al., 2004; Hooper et al., 2008; Hwang et al., 2012). Most importantly, polyphenolic flavonoids play a key neuroprotective role against various neurotoxic conditions and paradigms (Dajas et al., 2003; Gopinath et al., 2011; Scapagnini et al., 2011; Prakash et al., 2013; Prakash and Sudhandiran, 2015; Ahmad et al., 2016; Khan et al., 2016; Ali et al., 2018). Quercetin (3,5,7,3′,4′-pentahydroxyflavone) is a well-known natural flavonoid abundantly found in fruits and vegetables such as apples, berries, onions and capers; a normal human diet includes a daily intake of up to 25 mg of this compound. Quercetin possesses multiple forms of biological activity, including antitumoral, antithrombotic, anti-inflammatory and antiapoptotic activities (Dajas et al., 2003; Zhang et al., 2011; Costa et al., 2016). Quercetin exerts anti-inflammatory activity by inhibiting the proinflammatory cytokines that are released by glial cells. It has been reported that quercetin protects against neuroinflammation by inhibiting nitric oxide (NO) production in microglial cells, which further leads to the inhibition of NF-κB signals and prevents inflammatory-related neuronal injury (Chen W.W. et al., 2016; Rao et al., 2005; Kao et al., 2010; Liao and Lin, 2015). Similarly, quercetin ameliorated activated astrocytes and prevented zidovudine-induced neuroinflammation in the CNS (Yang et al., 2018). Activated astrocytes and microglia mediate the activation of cytokines and reactive oxygen species, which further affect neuronal cells and trigger the degeneration of neurons (Hong, 2017; Shal et al., 2018). It has been reported that quercetin attenuates manganese-induced neurotoxicity by preventing neuroinflammation-mediated neurodegeneration, which it accomplishes via regulating the heme oxygenase-1 (HO-1)/nuclear factor erythroid 2-related factor 2 (Nrf2) and nuclear factor kappa B (NF-kB) pathway (Bahar et al., 2017). Furthermore, it has been found that quercetin has a neuroprotective effect against neurodegeneration in various *in vitro* and *in vivo* mouse models (Bureau et al., 2008; Yang et al., 2014; Lei et al., 2015; Pogacnik et al., 2016; Shveta et al., 2016). The present study was conducted to explore the neuroprotective effect of quercetin against LPS-induced neuroinflammation-mediated neurodegeneration in the adult mouse cortex and hippocampus.

## Materials and Methods

### Mouse Strain, Housing and Ethical Considerations

Wild-type male C57BL/6N mice (age 8 weeks, body mass 25–30 g) were purchased from Samtako Bio (South Korea). The mice were acclimatized for 1 week in the university animal house under a 12-h/12-h light/dark cycle at 23°C with 60 ± 10% humidity and provided with food and water *ad libitum*. The maintenance and treatment of the mice were carried out in accordance with the Institutional Animal Care and Use Committee (IACUC) guidelines issued by the Division of Applied Life Science, Gyeongsang National University, South Korea. All efforts were made to minimize the suffering of animals. The experimental methods with mice were carried out in accordance with the approved guidelines (Approval ID: 125), and all experimental protocols were approved by the IACUC of the Division of Applied Life Science, Gyeongsang National University, South Korea.

### Animal Grouping and Treatments

**FIGURE 1:**
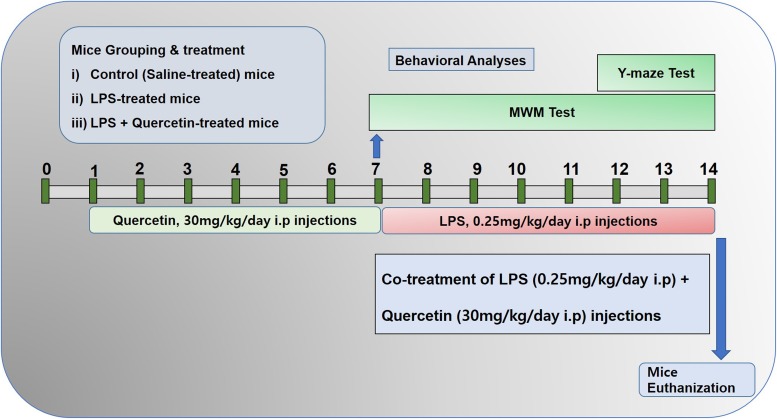
Schematic of the experimental schedule. (1) Control paradigm mice (Cont) treated with saline as vehicle for 2 weeks. (2) Mice treated with vehicle for 1 week and LPS 0.25 mg/kg/day for 1 week. (3) Mice treated with LPS 0.25 mg/kg/day for 1 week and quercetin 30 mg/kg/day for 2 weeks (1 week before the LPS and 1 week cotreated with LPS). After behavioral analyses on the 14th day, the adult mice were euthanized and further subjected to western blotting and morphological analyses.

The schematic presentation of animal grouping and treatment is indicated in Figure 1. After acclimatization, the mice were placed in the following groups: (1) Control mice injected with saline [intraperitoneally (I.P.)] as vehicle for 2 weeks; (2) Mice injected with LPS (0.25 mg/kg/day, I.P.) for 1 week; and (3) Mice injected with LPS (0.25 mg/kg/day, I.P.) for 1 week and quercetin (30 mg/kg/day, I.P.) for 2 weeks (1 week prior to the LPS and 1 week cotreated with LPS).

The dosage of quercetin was selected in accordance with previously reported studies that quercetin at a 30 mg/kg body weight dose induced more significant and beneficial effects than 10 or 20 mg/kg (Haleagrahara et al., 2009; Yang et al., 2014; Park et al., 2018). Quercetin was dissolved in dimethyl sulfoxide (DMSO) to prepare the stock solution. Each day, fresh quercetin solution was prepared in normal saline according to the required volume of injection (250 μl/mouse/day). LPS dissolved in normal saline, and the same volume was administered I.P. to the mice. Every day at the same time, the mice were brought to the injection room for the injections.

### Behavioral Study

To investigate the effect of quercetin on memory functions, we performed a behavioral study (*n* = 15/group) using a Morris water maze (MWM) task and a Y-maze task.

The MWM test is a parameter task to evaluate memory functions. The experimental apparatus consisted of a circular water tank (100 cm in diameter, 40 cm in height) containing water (23 ± 1°C) to a depth of 15.5 cm, which was rendered opaque by adding white paint. A transparent escape platform (10 cm in diameter, 20 cm in height) was hidden 1 cm below the water surface and placed at the midpoint of one quadrant. The MWM test was started on day 7 and completed on the 13th day of the experimental schedule (Figure 1). Each mouse received training each day for 6 consecutive days using a single hidden platform in one quadrant with three rotating starting quadrants. Latency to escape from the water maze (finding the submerged escape platform) was calculated for each trial. On day seven, final escape latency and probe tests were performed to evaluate memory consolidation. The probe test was performed by removing the platform and allowing each mouse to swim freely for 60 s. The time the mice spent in the target quadrant (where the platform was located during hidden platform training) was measured. The time spent in the target quadrant is considered to represent the degree of memory consolidation that has taken place after learning. All data were recorded using video-tracking software (SMART, Panlab Harvard Apparatus Bioscience Company, United States).

The Y-maze was built from wood that had been painted black. Each arm of the maze was 50 cm long, 20 cm high, and 10 cm wide at the bottom and top. The Y-maze was started on day 12 and completed on day 14 of the experimental schedule (Figure 1). Each mouse was placed at the center of the apparatus and allowed to move freely through the maze for three 8-min sessions. The series of arm entries was visually observed. Spontaneous alteration was defined as the successive entry of the mice into the three arms in overlapping triplet sets. Alteration behavior (%) was calculated as follows: [successive triplet sets (entries into three different arms consecutively)/total number of arm entries-2] × 100.

### Protein Extraction From Mouse Brain

After behavioral studies, all mice were brought to the surgical room and anesthetized with 0.05 ml/100 g body weight Rompun (Xylazine) and 0.1 ml/100 g body weight Zoletil (ketamine). After anesthesia, the mice were euthanized via decapitation, and brain tissue was immediately removed, and the cortex and hippocampus were separated and stored at −80°C. The cortical and hippocampal tissues were homogenized in PRO-PREP^TM^ protein extraction solution according to the manufacturer’s instructions (iNtRON Biotechnology, Inc.). The samples were then centrifuged at 13000 rpm at 4°C for 25 min. The supernatants were collected and stored at −80°C.

### Western Blot Analysis

Western blotting was performed as described previously (Ali et al., 2018). Briefly, the protein concentrations in the samples were measured (BioRad protein assay kit, BioRad Laboratories, CA, United States). Equal amounts of protein (15–30 μg) were electrophoresed on a 12–15% SDS-PAGE gel and transferred to a polyvinylidene difluoride (PVDF) membrane. A protein marker (GangNam-STAIN, iNtRON Biotechnology) was run in parallel for detection of the molecular weights of the proteins. To reduce the non-specific binding membrane, the membranes were blocked using 5% skim milk and incubated with primary antibodies anti-ionized calcium binding adapter molecule 1 (Iba-1), anti-glial fibrillary acidic protein (GFAP), anti-phosphorylated-nuclear factor kappa B (p-NF-kB) 65, anti-toll-like receptor-4 (TLR-4), anti-postsynaptic density protein (PSD)-95, anti-synaptophysin (Synap), anti-tumor necrosis factor-α (TNF-α), anti-nitric oxide synthase-2 (NOS-2), anti-cyclooxygenase-2 (COX-2), anti-caspase-3, anti-poly (ADP-ribose) polymerase-1 (PARP-1), anti- cytochrome c (Cyto. c), anti-Bax, anti-Bcl2, and anti-β-actin from Santa Cruz Biotechnology, Dallas, TX, United States, overnight at 4°C at 1:1000 dilution (Table 1). Immunoreaction was detected using chemiluminescence (Amersham ECL Advance Western Blotting Detection Reagent). The X-ray films were scanned, and the optical densities of the bands were measured using Computer-based Sigma Gel software (SPSS, Chicago, IL, United States).

**Table 1:** Primary antibodies information.

	Host	Application	Manufacturer	Catalog number	Concentration
Iba-1	Rabbit	WB	Santa Cruz Biotechnology, United States	SC 98468	1:1000
GFAP	Mouse	WB/IF	=	SC: 33673	1:1000/1:100
TLR-4	Goat	WB	=	SC: 16240	1:1000
p-NF-kB	Mouse	WB/IF	=	SC 8008	1:1000/1:100
TNF-α	Mouse	WB	=	SC: 8436	1:1000
COX-2	Rabbit	WB	=	SC:7951	1:1000
NOS-2	Rabbit	WB	=	SC:651	1:1000
IL-lβ	Mouse	IF	=	SC: 32294	1:100
Bax	Mouse	WB	=	SC: 7480	1:1000
Bcl-2	Mouse	WB	=	SC: 7382	1:1000
Cyto. c	Mouse	WB	=	SC: 13156	1:1000
PARP-1	Mouse	WB	=	SC: 8007	1:1000
Caspase-3	Mouse	WB/IF	=	SC: 7272	1:1000/1:100
PSD-95	Mouse	WB	=	SC:71933	1:1000
Synap	Rabbit	WB	=	SC: 17750	1:1000
SNAP-23	Mouse	IF	_=_	SC: 374215	1:100


### Brain Tissue Collection and Sample Preparation for the Immunohistofluorescence Staining

After behavioral studies, all mice were brought to the surgical room and anesthetized with 0.05 ml/100 g body weight Rompun (Xylazine) and 0.1 ml/100 g body weight Zoletil (ketamine). The mice were perfused transcardially with 0.9% normal saline solution and 4% paraformaldehyde. The mice were euthanized via decapitation, and brain tissue was immediately removed from all mice and fixed with ice-cold paraformaldehyde at 4°C for 72 h, then submerged in 20% sucrose phosphate buffer for 72 h. All brains were frozen in O.C.T. compound (A.O., United States) and then cut into 14-μm coronal sections using a CM 3050C cryostat (Leica, Germany). The sections were thaw mounted on ProbeOn Plus charged slides (Fisher, United States).

### Immunofluorescence Staining

The immunofluorescence staining proceeded as described previously with some modifications (Badshah et al., 2016). Briefly, slides containing brain sections were washed twice for 10 min each in 0.01 M PBS, 1X proteinase K was added to the tissue, and the slides were incubated at room temperature for 5 min. The slides were washed twice for 5 min each, followed by incubation for 1 h in blocking solution containing 2% normal serum and 0.3% Triton X-100 in 0.01 M PBS according to the antibody treatment. After the slides were blocked, they were incubated overnight at 4°C with primary antibodies GFAP, p-NF-kB, IL-1β, caspase-3 and SNAP23 from Santa Cruz Biotechnology, Dallas, TX, United States, diluted 1:100 in blocking solution. Following incubation with primary antibodies, the sections were incubated for 2 h in the secondary tetramethyl rhodamine isothiocyanate (TRITC)/fluorescein isothiocyanate (FITC)-labeled antibodies (1:50) (Santa Cruz Biotechnology, Dallas, TX, United States). After incubation with the TRITC/FITC-labeled antibodies, the slides were mounted with DAPI and Prolong Antifade Reagent. The images were captured using a FluoView FV 1000 laser confocal microscope equipped with FV10-ASW 3.1 Viewer (Olympus, Tokyo, Japan). The number of original confocal images per tissue was five per group, and the images were converted into TIF images. The fluorescence intensity of the same region of the cortex/total area and hippocampus/total area of the TIF images for all groups were measured using ImageJ software via the following method. The TIF image background was optimized according to the threshold intensity, and the immunofluorescence intensity, analyzed at the same threshold intensity for all groups, was expressed as the relative integrated density of the samples relative to the control.

### Nissl Staining

To analyze neuronal loss and survival, Nissl staining was used as previously described with minor changes (Ali et al., 2015b; Badshah et al., 2015a). In brief, all slide sections were washed twice for 10–15 min in PBS (0.01 M) and incubated in 0.5% cresyl violet solution containing a few drops of glacial acetic acids for 10–15 min. The tissues were washed with distilled water and dehydrated in graded ethanol (70, 95, and 100%). After graded dehydration, the tissues were placed in xylene twice for 3 min each. The tissues were covered with a coverslip using mounting medium. Immunohistochemical TIF images were captured with a fluorescence light microscope. The number of images per slide was five for each group. The immunohistochemical intensity for the number of surviving neurons in the cortex/total area and hippocampus/total area (CA1) of the brain was counted using ImageJ software via the following method. The TIF image background was optimized according to the threshold intensity and analyzed the survival neuronal cells at the same threshold intensity for all groups and was expressed as the relative integrated density for the number of surviving neurons of the samples relative to the control.

### Chemicals

LPS, quercetin [2-(3, 4-Dihydroxyphenyl)-3, 5, 7-trihydroxy-4H-1-benzopyran-4-one] and DMSO were purchased from Sigma-Aldrich Chemical Co. (St. louis, MO, United States).

### Data and Statistical Analyses

Western blot bands were scanned and analyzed by densitometric analyses using the Sigma Gel System (SPSS, Chicago, IL, United States). ImageJ software (National Institutes of Health, Bethesda, MD, United States) was used for the densitometric analyses of the immunofluorescence and immunohistofluorescence images. All histograms were made using GraphPad Prism 5/6 (GraphPad Software, San Diego, CA, United States). For comparisons among the treatment groups and the control groups, statistical analyses were performed using one-way analysis of variance (ANOVA) followed by a two-tailed independent Student’s *t-test* and Tukey’s multiple comparison test where appropriate. The expressed data are presented as the means ± SEM of the three independent experiments. Statistical significance = *P < 0.05*^∗^. Significantly different compared with the control group; **#** significantly different compared with the LPS-injected mice.

## Results

### Quercetin Improved the Memory Function of the LPS-Injected Mice

**FIGURE 2:**
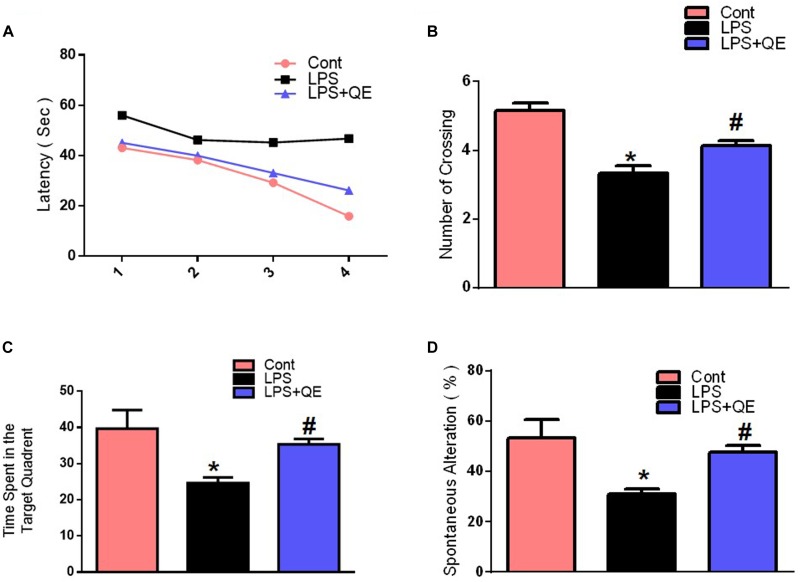
Quercetin improved the memory function of the LPS-treated mice. For the behavioral analyses, the MWM and Y-maze tests were used to investigate and evaluate the memory functions of the control, LPS and LPS+quercetin group mice. **(A)** Average escape latency time for experimental mice to reach the hidden platform from 1 to 4 days. **(B)** The average number of crossings at the hidden platform during the probe test of the MWM test. **(C)** Time spent in the platform quadrant, where the hidden platform was placed during the trial session. **(D)** Spontaneous alteration behavior % of the mice during the Y-maze test. Histograms indicate the means ± SEM for the mice (*n* = 15/group). ^∗^ Significantly different from the control; # significantly different from LPS-treated group. Significance: *P* < 0.05.

Mounting studies have supported the evidence that natural-derived substances, particularly flavonoids, have a promising role in the enhancement of learning, and memory functions (Scapagnini et al., 2011; Prakash et al., 2013; Liu et al., 2014; Ahmad et al., 2016; Ali et al., 2018). Quercetin also has a beneficial effect on memory and cognitive functions (Sriraksa et al., 2012; Ashrafpour et al., 2015). However, numerous studies have investigated that systemic LPS administration induces memory and cognitive dysfunction (Qin et al., 2007; Badshah et al., 2016; Khan et al., 2016, 2017). Therefore, to assess the memory-enhancing effect of quercetin against systemic LPS, we designed a dosage regimen of quercetin at a 30 mg/kg body weight dose for 2 weeks (1 week prior and 1 week cotreated with LPS) via the I.P route. Other studies also recommended a quercetin dose of 30 mg/kg/day I.P. for a short period of time induce beneficial effects (Haleagrahara et al., 2009; Yang et al., 2014; Park et al., 2018). We evaluated the memory functions of the mice using MWM and Y-maze tests. Initially, we trained all animals in an MWM task where they were required to find a submerged hidden platform and then analyzed the time required to reach the hidden platform. The LPS-injected animals took more time to find the hidden platform compared to the control mice (Figure 2A). However, quercetin treatment reversed the LPS effect and significantly improved memory function, as indicated by the animals taking less time to reach the hidden platform compared to the LPS-injected mice. Furthermore, a probe test showed that quercetin reversed the LPS effect and led to a significant increase in the number of platform crossings and an increase in the time spent in the target quadrant in which the hidden platform was previously located (Figures 2B,C). These results demonstrated that quercetin reversed the detrimental effect of LPS and significantly improved memory performance.

The Y-maze results also indicated that LPS triggered short-term spatial memory dysfunction compared to the control group. Quercetin treatment significantly enhanced the spontaneous alteration behavior percentage (a parameter for the enhancement of spatial working memory functions), indicating that quercetin improved the spatial working memory function of the LPS-injected mice (Figure 2D).

### Quercetin Protects Against LPS-Induced Synaptic Dysfunction

**FIGURE 3:**
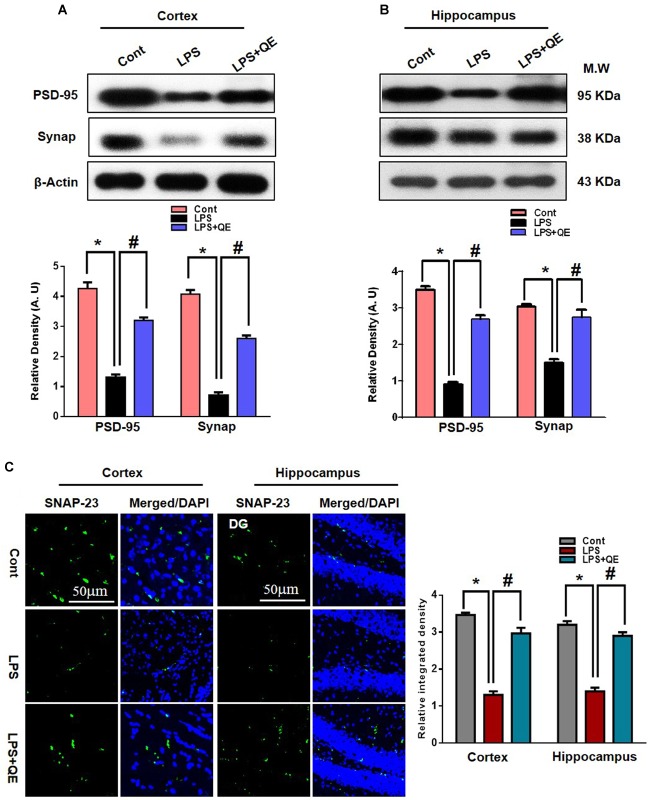
Quercetin improved the pre- and postsynaptic markers in the LPS-treated mice. **(A,B)** Western blotting of the proteins Synap and PSD95; their differences in the cortex and hippocampus of mouse brains are represented by a histogram. β-Actin was used as a loading control. The quantified density values are shown in arbitrary units (A.U.) as the means ± SEM for the respective shown protein (8 mice/group). **(C)** Representative immunofluorescence images and the quantified histogram of SNAP-23 in the cortex and hippocampus of adult mice (5 mice/group). Magnified 10×. Scale bar = 50 μm. The expressed data are relative to the control. ^∗^ Significantly different from the control; # significantly different from LPS-treated group. Significance: *P* < 0.05.

Mounting studies have reported that flavonoids are beneficial for synaptic and memory functions (Ahmad et al., 2016; Matias et al., 2017; Ali et al., 2018; Khan et al., 2018). Because synaptic (pre- and postsynaptic) proteins have been associated with the decline of memory and cognitive functions. Therefore, we also examined the effects of quercetin on synaptic expression levels by western blot and confocal microscopy. The western blot (Figures 3A,B) results show that the group of mice that received LPS had decreased expression levels of PSD-95 and Synap in the cortex and hippocampus compared to the control group of adult mice. Treatment with quercetin along with LPS significantly reversed the LPS-induced synaptic deficit by increasing the expression of PSD95 and Synap in the cortex and hippocampus of adult mice (Figures 3A,B). To further verify the effect of quercetin on synaptic function, we examined SNAP-23 expression levels using confocal microscopy. The immunofluorescence images showed reduced reactivity in the LPS-treated mice compared to the control group. Quercetin treatment significantly increased the immunofluorescence reactivity of SNAP-23 in the cortex and DG region of the hippocampus compared to the LPS-treated group of adult mice (Figure 3C).

### Quercetin Attenuates the LPS-Induced Activation of Microglia and Astrocytes

**FIGURE 4:**
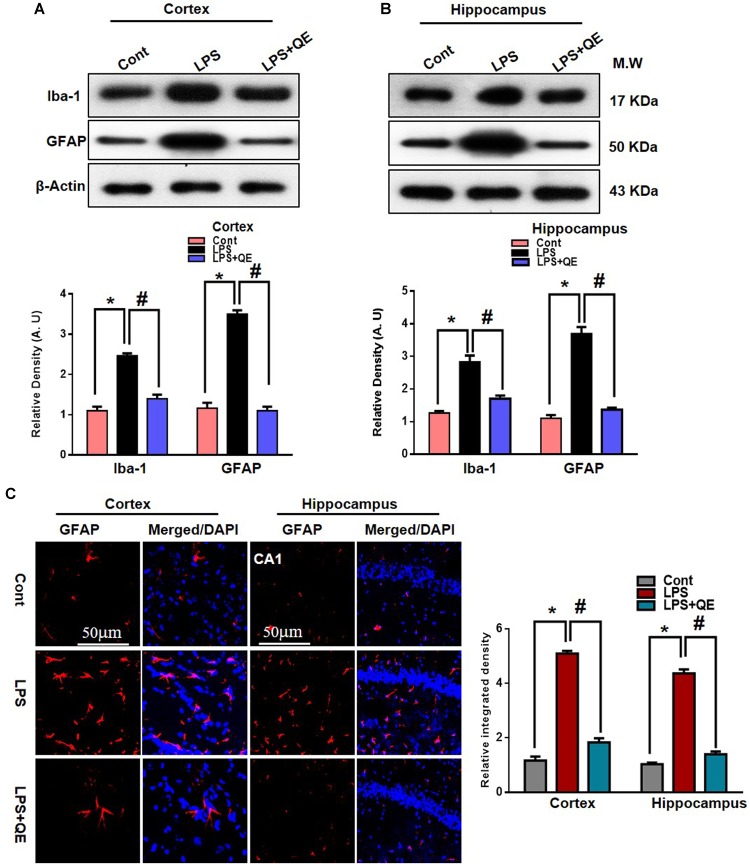
Quercetin ameliorated LPS-induced activated gliosis in the cortex and hippocampus of adult mice. **(A,B)** Western blotting of the proteins GFAP and Iba-1; their differences in the cortex and hippocampus of mouse brains are represented by a histogram. β-Actin was used as a loading control. The quantified density values are shown in arbitrary units (A.U.) as the means ± SEM for the respective shown protein (8 mice/group). **(C)** Representative immunofluorescence images and the quantified histogram of activated GFAP in the cortex and hippocampus of adult mice (5 mice/group). Magnified 10×. Scale bar = 50 μm. The expressed data are relative to the control. ^∗^ Significantly different from the control; # significantly different from LPS-treated group. Significance: *P* < 0.05.

Microglia and astrocytes in the CNS respond rapidly to complaints such as infections, stress, and injury, which makes them important modulators of neuroinflammation responses (Witte et al., 2010; Chen et al., 2012; Badshah et al., 2015b). Studies have reported that in systemic LPS administration, activated microglia and astrocytes are responsible for neuroinflammation-mediated neurodegeneration (Qin et al., 2007; Badshah et al., 2015b, 2016; Khan et al., 2016; Rehman et al., 2018). GFAP protein and Iba-1 are specific markers for activated astrocytes and microglia, respectively. Flavonoids, on the other hand, have been reported to have multiple neuroprotective properties, including potent anti-inflammatory effects. Quercetin, a natural flavonoid, also shows a strong anti-inflammatory action by suppressing activated astrocytosis and microgliosis (Chen et al., 2005; Bureau et al., 2008; Kao et al., 2010; Rinwa and Kumar, 2013; Yang et al., 2018). To analyze the expression of GFAP and Iba-1, we found through western blotting that LPS treatment significantly increased the expression of these two proteins in the adult mouse cortex and hippocampus compared to the control group of mice. Treatment with quercetin along with LPS significantly decreased the expression of these proteins in the adult mouse cortex and hippocampus (Figures 4A,B). In addition, the GFAP expression level was also analyzed using immunofluorescence staining. The immunofluorescence results showed the increased intensity of GFAP-positive cells and immunofluorescence reactivity in the LPS-treated group compared to the control groups. Quercetin treatment along with LPS significantly reduced the GFAP-positive cells and immunofluorescence reactivity compared to LPS-treated mice (Figure 4C).

### Quercetin Halts the LPS-Induced Activated TLR4/NFKB Pathway

**FIGURE 5:**
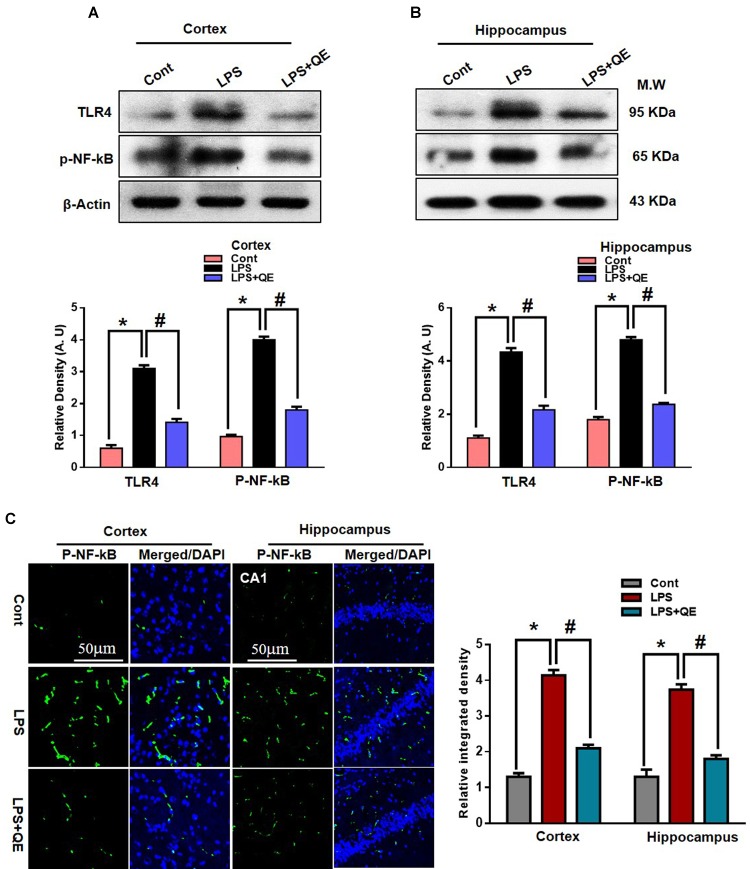
Quercetin halts the LPS-induced activated TLR4/NFKB pathway. **(A,B)** Western blotting of TLR4 and p-NFKB proteins; their differences in the cortex and hippocampus of mouse brains are represented by a histogram. β-Actin was used as a loading control. The quantified density values are shown in arbitrary units (A.U.) as the means ± SEM for the respective shown protein (8 mice/group). **(C)** Representative immunofluorescence images and the quantified histogram of p-NFKB in the cortex and hippocampus of adult mice (5 mice/group). Magnified 10×. Scale bar = 50 μm. The expressed data are relative to the control. ^∗^ Significantly different from the control; # significantly different from LPS-treated group. Significance: *P* < 0.05.

Mounting studies have demonstrated that LPS is known to activate microglia in several animal models, which leads to neuroinflammation and neurodegeneration (Johansson et al., 2005; Badshah et al., 2016; Carvalho et al., 2017; Khan et al., 2017; Jung et al., 2017). TLR-4 is a primary receptor for LPS-activated microglia (Qin et al., 2006; Badshah et al., 2016; Rehman et al., 2018). Here, we also found through western blotting that systemic administration of LPS activated TLR-4 in the adult mouse cortex and hippocampus compared to the control group of mice. Quercetin treatment along with LPS significantly decreased the expression of TLR-4 in the mouse cortex and hippocampus compared to LPS-treated mice (Figures 5A,B). Activated TLR-4 is responsible for inflammatory signaling in the MyD88-dependent pathway, which is responsible for the up-regulation of p-NF-κB and ultimately leads to neuroinflammation and neurodegeneration (Yao et al., 2017). We also found through western blotting that LPS administration activated p-NF-κB expression in the cortex and hippocampus of adult mice compared to the control group of mice. Treatment with quercetin significantly reduced the expression of p-NF-κB in the cortex and hippocampus of adult mice (Figures 5A,B).

Furthermore, the immunofluorescence results of p-NF-κB showed immunofluorescence reactivity in the LPS-treated group compared to the control groups. Quercetin treatment along with LPS significantly reduced the immunofluorescence reactivity compared to LPS-treated mice (Figure 5C).

### Quercetin Attenuated LPS-Induced Neuroinflammation-Associated Markers

**FIGURE 6:**
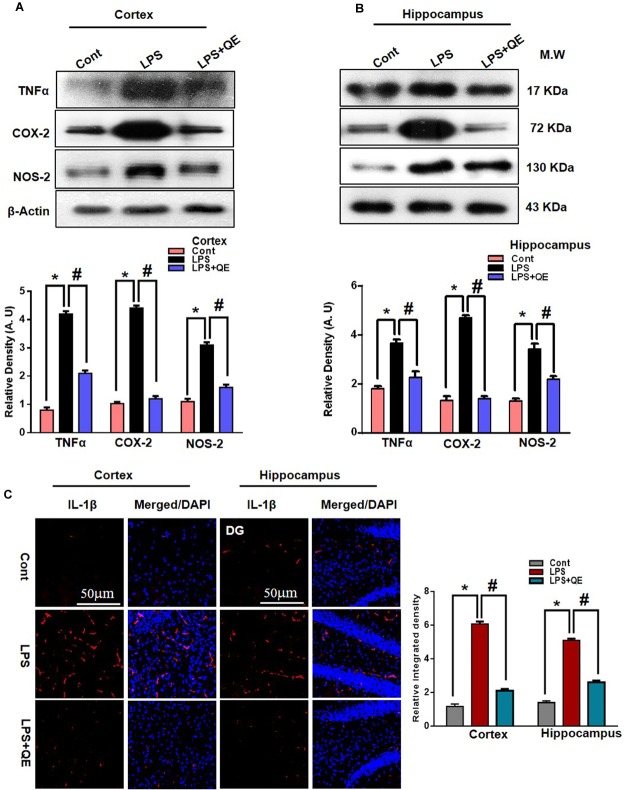
Quercetin attenuated LPS-induced neuroinflammation-associated markers. **(A,B)** Western blotting of TNF-α, COX-2 and NOS2 proteins; their differences in the cortex and hippocampus of mouse brains are represented by a histogram. β-Actin was used as a loading control. The quantified density values are shown in arbitrary units (A.U.) as the means ± SEM for the respective shown protein (8 mice/group). **(C)** Representative immunofluorescence images and the quantified histogram of IL-1β in the cortex and hippocampus of adult mice (5 mice/group). Magnified 10×. Scale bar = 50 μm. The expressed data are relative to the control. ^∗^ Significantly different from the control; # significantly different from LPS-treated group. Significance: *P* < 0.05.

Quercetin is a natural flavonoid found in many vegetables and fruits and possesses potential biological and health beneficial effects that have the ability to inhibit inflammatory mediators (Lesjak et al., 2018). It has been reported that LPS administration has the potential to increase the production of several inflammatory mediators, such as TNF-α, COX-2, NOS-2, and IL-1β (Badshah et al., 2015b, 2016; Khan et al., 2016, 2017). Here, we also ascertained through western blotting that LPS administration increased the expression of TNF-α, COX-2, and NOS-2 in the cortex and hippocampus of adult mice compared to the control group of mice. Quercetin treatment along with LPS significantly tempered and reduced the expression of these inflammatory proteins (Figures 6A,B). Similarly, the immunofluorescence results of IL-1β showed that LPS administration increased the number of IL-1β-positive cells compared to control mice in the cortex and DG region, but the group of mice that received quercetin along with LPS significantly downregulated the IL-1β-positive cells and fluorescence immunoreactivity in the cortex and DG region (Figure 6C).

### Quercetin Prevented LPS-Induced Neuronal Degeneration

The mitochondrial apoptotic pathway plays a key role in neuronal degeneration. In the mitochondrial apoptotic pathway, the antiapoptotic Bcl-2 and proapoptotic Bax markers have a primary role in the apoptotic pathway. The Bax/Bcl-2 ratio is an important indicator of the apoptotic pathway. Furthermore, increased Bax/Bcl-2 ratio induced overactivation of Cyto. c, an important mediator in the mitochondrial associated pathway, which leads to activation of caspases (Li et al., 1997; Chao and Korsmeye, 1998; Debatin et al., 2002; Badshah et al., 2015a). Previous studies (Badshah et al., 2015a; Khan et al., 2016) have indicated that flavonoids play a key role in regulating the mitochondrial apoptotic pathway; therefore, we also investigated the effect of quercetin on the mitochondrial pathway. We ascertained through western blots that LPS triggers the Bax/Bcl-2 ratio and release of Cyto. c compared to the control group. Quercetin significantly reduced the Bax/Bl2 ratio and Cyto. c expression level compared to the LPS-treated group alone.

**FIGURE 7:**
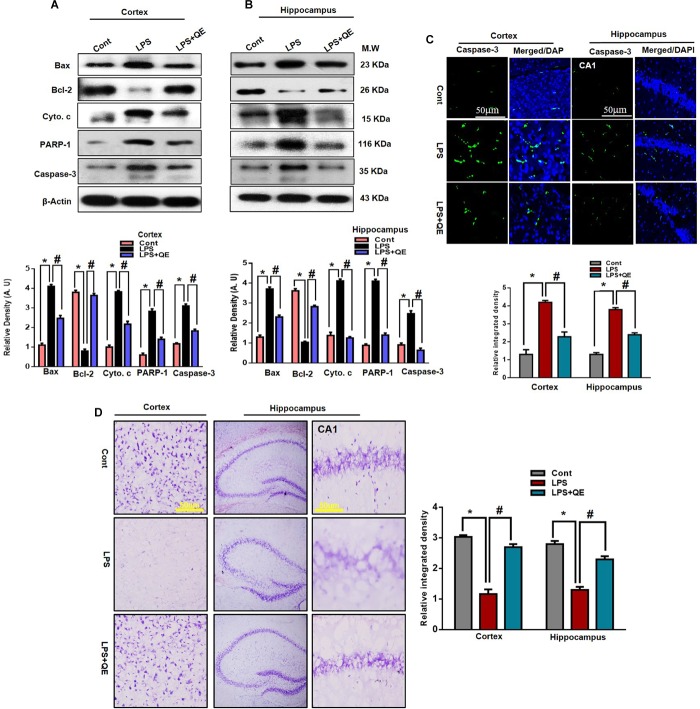
LPS-induced neuronal apoptotic pathway prevented by quercetin in the cortex and hippocampus. **(A,B)** Western blotting of Bcl-2, Bax, Cyto. c, Caspase-3 and PARP-1 proteins; their differences in the cortex and hippocampus of mouse brains are represented by a histogram. β-Actin was used as a loading control. The quantified density values are shown in arbitrary units (A.U.) as the means ± SEM for the respective shown protein (8 mice/group). (**C**) Representative immunofluorescence images and the quantified histogram of Caspase-3 in the cortex and hippocampus of adult mice (5 mice/group). Magnified 10×. Scale bar = 50 μm. (**D**) Representative immunohistochemical images (Nissl staining) and the quantified histogram of the survival neuron reactivity and integrated density in the cortex and hippocampus region of adult mice. The expressed data are relative to the control. ^∗^ Significantly different from the control; # significantly different from LPS-treated group. Significance: *P* < 0.05.

In apoptotic neurodegeneration, the caspase family plays an important role. Among caspase cascades, caspase-3 is the major player in apoptosis and plays a key role in apoptosis (Le et al., 2002; Carloni et al., 2004; Ali et al., 2015a; Badshah et al., 2015a, 2016). Therefore, we also evaluated caspase-3 activity by western blotting and confocal microscopy. Our western blot results show that LPS activated caspase-3 activity in the cortex and hippocampus of adult mice compared to the control group of mice. Treatment with quercetin along with LPS significantly reduced the activated caspase-3 activity compared to LPS-treated mice (Figures 7A,B). Similarly, the confocal microscopy results showed that there are more caspase-3-positive cells and fluorescence reactivity of caspase-3 in the cortex and CA-1 region of the LPS-received mice compared to the control group of mice. However, treatment with quercetin significantly reduced caspase-3-positive cells and fluorescence immunoreactivity in the cortex and CA-1 region of the brain (Figure 7C).

PARP-1is a nuclear enzyme with a wide range of physiological and pathological functions. In physiological function, it is involved in DNA repair and genomic stability. In pathological conditions, the over activation of PARP-1 leads to neuronal cell death (Berger, 1985; Chaitanya et al., 2010). It has been reported that activated caspase-3 increased the over activation of PARP-1 (Williams et al., 2008; Badshah et al., 2015a). Therefore, in this context, we also evaluated the expression of PARP-1 through western blotting in both the cortex and hippocampus of adult mice. Our western blotting results revealed that systemic LPS administration results in the overexpression of PARP-1 in the cortex and hippocampus of adult mice compared to the control group of mice. Treatment with quercetin along with LPS reduced the expression of PARP-1 in the adult mouse cortex and hippocampus (Figures 7A,B). Furthermore, the immunohistochemical Nissl staining results showed that LPS injection decreased the neuronal survival reactivity in the cortex and hippocampus of adult mouse brains compared to the control group of mice. Importantly, quercetin administration to LPS-injected mice enhanced the survival of neuronal cells in the cortex and hippocampus of adult mouse brains (Figure 7D).

## Discussion

The application and consumption of natural substances is a primary focus for the prevention of neurodegenerative diseases. Among natural substances, polyphenol-derived medicinal substances are important therapeutic agents for the slowing or prevention of neurological disorders (Dajas et al., 2003; Scapagnini et al., 2011; Prakash et al., 2013; Liu et al., 2014; Badshah et al., 2015a; Ahmad et al., 2016; Jung et al., 2017; Ali et al., 2018). Quercetin is a well-approved and recommended flavonoid that has medicinal properties and protective roles in different paradigms of CNS insult-induced detrimental effects (Lei et al., 2015; Chen S. et al., 2016; Kanter et al., 2016). In this study, we also investigated the neuroprotective effect of quercetin against LPS-induced detrimental effects such as neuroinflammation-mediated neurodegeneration and synaptic/memory deficits in the cortical and hippocampal regions of the adult mouse brain.

Chronic neuroinflammation is a pathological cascade that occurs during the progression of several neurological disorders, such as AD, PD, FTD, and amyotrophic lateral sclerosis (ALS) (Glass et al., 2010; Von Bernhardi et al., 2010; Chen W.W. et al., 2016; Hong, 2017). In chronic neuroinflammation, activated microglia and astrocytes disturb homeostasis and are implicated in all degenerative conditions of the CNS (Netea et al., 2003; Perry et al., 2003; Hoogland et al., 2015; Jung et al., 2017). Previous studies have shown that systemic administration of LPS activates microglia and astrocytes (Qin et al., 2007; Badshah et al., 2016). The TLR family has a promising and key role in the immune response. This family comprises 13 members in rodents and 11 members in humans. Furthermore, several studies have confirmed that TLR-4 is a primary target and receptor in glial cells (Shimazu et al., 1999; Aravalli et al., 2007; Block et al., 2007; Glass et al., 2010; Rehman et al., 2018). In both *in vivo* and *in vitro* evidence confirmed that LPS binds to TLR-4, inducing activated gliosis, which consequently mediates NF-kB cascade activation, which plays a serious role in the activation of inflammation and neurodegeneration processes (Chen et al., 2012; Catorce and Gevorkian, 2016). NF-kB has been considered a mediator between neuroinflammation and neurodegeneration. Several studies reported that natural flavonoids prevented activated gliosis by inhibiting completely or partially by inhibiting the TLR4 and NF-kB cascades (Lee et al., 2012; Badshah et al., 2016; Khan et al., 2016; Rehman et al., 2018). The inhibition of the TLR4 and NF-kB cascades confers desirable effects in any pathogenic and neurotoxic condition. Bureau et al., 2008, reported that quercetin inhibited LPS-induced activated glial cells. Likewise, we have found that quercetin administration prevents LPS-induced activated gliosis by reducing the expression of TLR4 and NF-kB cascades.

Activated microglia and astrocytes are responsible for the release of inflammatory molecules such as TNF-α, IL-1β, COX-2, and NOS2, which are responsible for neuroinflammation. Studies have reported that activated nuclear translocation of the NF-kB cascades pathway is implicated in the over production and release of the above proinflammatory mediators (Li and Verma, 2002; Lee et al., 2012; Song et al., 2014; Gu et al., 2015; Hong, 2017). In a literature review reported that transgenic rodents that overexpressed TNF-α exhibited inflammation and neurodegeneration, which lead to memory impairment. Over activation of TNF-α has been reported to induce neurotoxicity in human cortical neurons. Similarly, mounting studies have reported overexpressed immunoreactive IL-1β cells in pathogenic conditions, brain injuries and degeneration. Overexpressed IL-1β affects both neuronal and non-neuronal cells in the CNS Wyss-Coray and Rogers (2012). In addition, when murine BV2 microglial cells are exposed to LPS- and IFN-γ-induced NO production and iNOS gene expression, neuroinflammation-mediated neurodegeneration is triggered (Chen et al., 2005; Song et al., 2014). Interestingly, quercetin acts as an antioxidant and anti-inflammatory agent to inhibit NO and iNOS expression by regulating the NF-kB/HO pathway in LPS-exposed BV2 cells (Chen et al., 2005; Kao et al., 2010). TNF-α, IL-1β and reactive species such NO and iNOS induced the overexpression of COX2, which has a key role in the intensification of neuroinflammation-mediated neurodegeneration (Feng et al., 1995; Yamamoto et al., 1995; O’Banion et al., 1996; Salvemini, 1997). Recent attention has been given to natural compounds such as flavonoids that possess multiple neuroprotective activities, such as suppressing neuroinflammation and neuronal apoptosis, and promoting neuronal survival and memory enhancing effect (Lee et al., 2012**;** Dey et al., 2017; Shal et al., 2018). Flavonoids have been suggested as promising therapeutic agents for the reduction of neuroinflammation (Magalingam et al., 2015; Chen S. et al., 2016). Quercetin is found abundantly in onions and various berries. Studies have reported that quercetin shows strong activity against neuroinflammation (Spencer, 2008; Kanter et al., 2016; Matias et al., 2016; Duet al., 2016). In the present study, our results supported the previous findings and elucidated that quercetin suppressed the proinflammatory mediators as described above and consequently attenuated LPS-induced neuroinflammation in the adult mouse cortex and hippocampus.

Chronic neuroinflammation mediates the neuronal degeneration process in various diseases, such as AD, PD, and ALS. In both *in vivo* and *in vitro* studies, LPS-induced activated cytokines and chemokines as well as activated redox and nitrogen species, which further trigger apoptotic neurodegeneration (Li and Verma, 2002; Chen et al., 2005; Chen W.W. et al., 2016; Kao et al., 2010; Lee et al., 2012; Song et al., 2014; Gu et al., 2015; Kempuraj et al., 2016, 2017; Hong, 2017). Studies have reported that LPS induces the mitochondrial apoptotic pathway by interfering with Bax/Bcl-2 signaling (Badshah et al., 2015b, 2016; Khan et al., 2016, 2017). Activated Bax/Bcl-2 triggers the activation of Cyto. c, which further triggers the activation of caspase cascades. Caspase cascades, e.g., caspase-3, play a major role in apoptotic neuronal degeneration. The activation of caspase-3 induced neuronal cell death and has been considered a main feature of neurodegenerative diseases. Activated caspase-3 cleaves PARP-1, which leads to neuronal DNA damage (Le et al., 2002; Carloni et al., 2004; Ali et al., 2015a; Badshah et al., 2015a, 2016). The natural dietary flavonoid shows a protective role against CNS-insult-induced neurodegeneration. Quercetin is a natural flavonoid that inhibits neuronal apoptotic cell death (Bureau et al., 2008; Yang et al., 2014; Lei et al., 2015; Kanter et al., 2016; Shveta et al., 2016). Interestingly, quercetin also regulated the mitochondrial apoptotic pathway and prevented the activation of Cyto. c, activated caspase 3 and cleaved PARP-1 expression and subsequently prevents neuronal degeneration, demonstrating that neuroinflammation-mediated neurodegeneration is rescued by quercetin.

It has been studied that systemic administration of LPS triggers neuroinflammation-mediated neurodegeneration, which is responsible for synaptic and memory dysfunction (Qin et al., 2007; Lee et al., 2012; Badshah et al., 2016). Flavonoids have been investigated well for improving learning and memory functions in aberrant and detrimental conditions (Scapagnini et al., 2011; Prakash et al., 2013; Liu et al., 2014; Ahmad et al., 2016; Ali et al., 2018). Studies show that LPS administration is responsible for decreasing the level of presynaptic and postsynaptic proteins (Badshah et al., 2016; Khan et al., 2018; Rehman et al., 2018). Our results also claimed that the systemic administration of LPS decreased the level of presynaptic proteins synaptophysin and postsynaptic protein PSD-95 in the mouse cortex and hippocampus. Our results show that quercetin treatment alleviates the LPS-induced impairment of synaptic functions in the mouse cortex and hippocampus. Similarly, we also observed that systemic LPS administration induced memory dysfunction. This memory dysfunction in the LPS-treated mice was reversed by quercetin, indicating that quercetin would be beneficial to improve the memory functions associated with synaptic functions in CNS-insult-induced detrimental effects.

In conclusion, our results demonstrated that quercetin prevented LPS-induced detrimental effects, such as neuroinflammation-mediated neurodegeneration and synaptic/memory impairment, in adult mice. These results suggest that drugs of natural origin with significant potential biological activity would be beneficial against pathogenic and neuronal insults in neurological disorders.

## Author Contributions

AK designed and managed the experimental work, and wrote the manuscript. TA contributed in the manuscript writing. AK, HB, SR, SA, KS, MI, TM, and MSK performed the western blot and morphological experiments. MOK was the corresponding author, having reviewed and approved the manuscript, and holds all the responsibilities related to this manuscript. All authors reviewed the manuscript.

## Conflict of Interest Statement

The authors declare that the research was conducted in the absence of any commercial or financial relationships that could be construed as a potential conflict of interest.
